# Simplifying phrases in depression screens: Interpreters’ views on usefulness in six languages

**DOI:** 10.1371/journal.pone.0292365

**Published:** 2023-12-08

**Authors:** Ulla Vanhatalo, Camilla Lindholm

**Affiliations:** 1 Department of Languages, Faculty of Arts, University of Helsinki, Helsinki, Finland; 2 Faculty of Information Technology and Communication Sciences, Communication Studies, Tampere University, Tampere, Finland; University of Florida, UNITED STATES

## Abstract

Assessments and treatments of mental health conditions such as depression use language that may be difficult to understand or translate. Here, we studied how interpreters assessed the usefulness of simplifying the language of a depression screen. Two alternative versions of the PRIME-MD PHQ depression screen were generated, with increasing linguistic simplicity. All the versions (standard, moderately simplified and most simplified) were translated from Finnish into Arabic, English, Farsi, Russian, and Swedish. Ten professional interpreters then assessed the different usefulness aspects of the three versions. The interpreters’ perceptions of usefulness of the different language versions for clients varied, and both simplified versions of the depression screen were commonly considered useful for interpreting contexts. The usefulness of the simplified language versions was seen as especially important for clients with multiple linguistic challenges, caused by, for example, dementia and immigration backgrounds. The language of depression screens can be greatly simplified. Simplified versions may significantly improve the accessibility of questionnaires for the wide range of individuals with compromised language competence. Simplified versions may also be helpful for inter-language interpreting in health care, and importantly, they may facilitate the transparency and cross-cultural calibration needed in evidence-based medicine.

## Introduction

Language is a key tool in all health and social care, and is particularly central for the diagnosis, assessment, and treatment of mental disorders. Most diagnostics of mental disorders are based on psychometric assessments, i.e., questionnaires and/or interviews that consist of internationally standardized sets of questions. These verbal questionnaires are typically constructed by collating phrases that health care workers consider useful. Interestingly, little has been done to verify the linguistic accessibility of the test items, despite it being well known that people with mental disorders may frequently present with compromised linguistic competence due to related medical or social conditions. For instance, people with immigrant backgrounds are at a higher risk of mental health conditions, especially depression and anxiety symptoms [1].

Previous works have shown that psychometric assessments of people with immigrant backgrounds may need a substantially different approach to those used with the native population [2–5]. Diagnostic screens may either explicitly or implicitly use cultural concepts and abstract language that are hard to comprehend in any language, or contain aspects that may have no appropriate equivalents in other languages [3, 5–7]. Such challenges in explaining and translating the meanings of words and/or concepts are well recognized in the linguistic field of lexical semantics.

Previous studies have shown that individuals with intellectual disabilities may benefit from adaptations to the language used [8]. A comparative study on linguistically adapted depression screens found that the Glasgow Depression Scale for people with a Learning Disability (GDS–LD, [9]) was the most promising for self-reporting [10]. Simplified versions of consent forms [11–13] and health leaflets [14] were also generally well received by patients with linguistic challenges. Surprisingly, however, the simplified and/or illustrated versions were not perceived as more understandable than the corresponding standard language versions [14–16]. Hence, the level of language simplification in prior studies may not have been sufficient to reach its key target––better linguistic comprehensibility.

The rapidly increasing demand for radically simplified language is widely recognized in society, and has given rise to the new field of Easy Language and accessible communication. As a result, Easy Language research in several European countries has set national guidelines for defining formal levels of Easy Language varieties according to their presumed target audiences with different language abilities [17, 18]. The simplest possible linguistic units required for communication have been studied a great deal in the field of Natural Semantic Metalanguage (e.g., [19, 20]). Extensive cross-linguistic studies have examined, for instance, emotion words, pain, and emotional connection, and have analyzed, discussed, and explained them using universal metalanguage (e.g., [21, 22]).

This study aimed to test the idea that increasing language simplicity in depression screens is beneficial when the text needs to be translated across languages and when the language competence of the target audience is compromised. To this end, we created two increasingly simplified versions of the PRIME-MD PHQ two-question depression screen, which were translated to five other languages. Then, we conducted a survey with ten professional interpreters to assess how well the different source and target versions could be received by interpreters or their clients with different competence profiles.

## Material and methods

**Fig 1:**
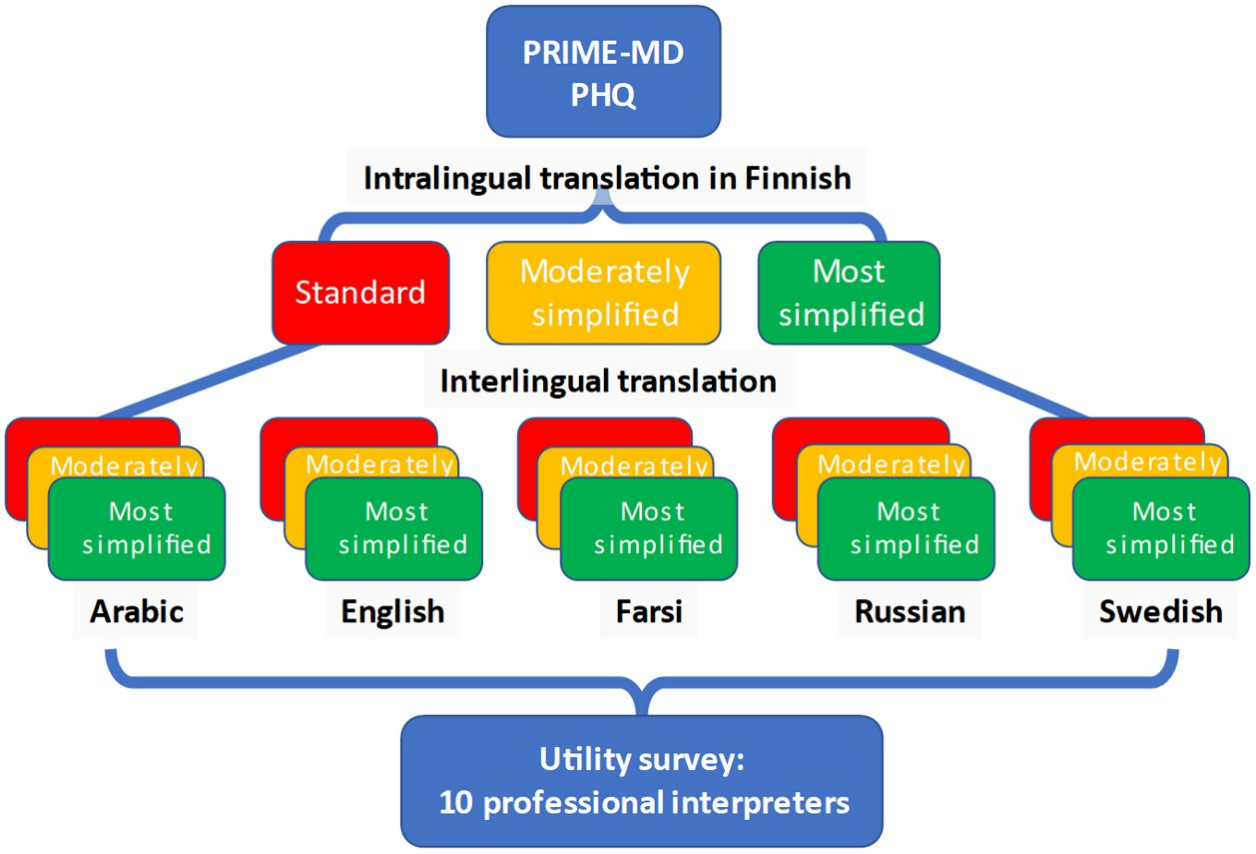
Overview of study rationale.

Fig 1 presents a schematic overview of the study rationale. In brief, we started with the original Finnish language version of the PRIME-MD PHQ depression screen, which we translated into two incrementally simpler language versions––moderately simplified and the most simplified. All three versions were then translated, by a commercial translation service, into five other languages––Arabic, English, Farsi, Russian, and Swedish. These languages were chosen because they are all commonly encountered in the Finnish health care system, and they represent different language groups and cultural backgrounds. Finally, we hired a group of ten professional interpreters with health care experience to participate in a survey to judge the various language versions in different contexts. These interpreters were blind to the previous steps in the present study, and were chosen for their language profile (two for each target language) and their prior experience in interpreting in health care settings.

### PRIME-MD PHQ two-question depression screen

The PRIME-MD PHQ two-question depression screen was chosen for this study for its brevity and compactness. Despite its seemingly straightforward appearance, its sentences are packed with abstract, culture-dependent vocabulary (*to bother*, *depressed*, *to feel down*, *hopeless*, *interest*, *pleasure*), which are likely to challenge translations into other languages and the understanding of individuals with any linguistic challenges. We are aware of that there may be other screens more accurate in general and in certain cultural contexts. Also, there may be tools originally written in easier language than PRIME-MD, and/or incorporating images. As for piloting and testing the idea of simplifying language, we needed a short and compact screen. For further studies, a longer screen or even a selection of different screens and screens including illustrations would be useful.

The PRIME-MD PHQ depression screen consists of two questions, and is the screening method in primary health care [23]. A patient is considered to need further examination if they respond positively to either of the questions. The official Finnish language version is openly available and recommended by the Finnish Current Care Guidelines website [24]. It is considered equivalent to the original English PRIME-MD PHQ two-question depression screen (see Table 1 for a comparison of the Finnish and English versions).

**Table 1:** PRIME-MD PHQ two-question depression screen in standard English and Finnish.

1. During the past month, have you often been bothered by feeling down, depressed, or hopeless? 2. During the past month, have you often been bothered by little interest or pleasure in doing things?	1. Oletko viimeisen kuukauden aikana usein ollut huolissasi tuntemastasi alakulosta, masentuneisuudesta tai toivottomuudesta? 2. Oletko viimeisen kuukauden aikana usein ollut huolissasi kokemastasi mielenkiinnon puutteesta tai haluttomuudesta?

### Linguistic simplifications

Linguistic complexity can be modified to different levels (vocabulary, syntax, information structure, content) and degrees. Here, we aimed to modify the degree of simplicity by exploiting linguistic norms that are well-established in the Finnish language: First, we took the standard Finnish version of official health care sources. Second, we translated it into *Easy Language* (also *Easy Read* or *easy-to-read*, and e.g., *Easy Finnish*) which is a widely used yet elusive concept involving attempts to render language easier to read or comprehend (e.g., [17, 18, 25]). Easy Language primarily aims to help people with compromised linguistic competence, such as people with immigration backgrounds, memory disorders or other cognitive disabilities. Easy Language production is viewed from the recipient’s perspective, and it tries to maximize comprehensibility by using simple, frequent vocabulary; by explaining any complex concepts; and by avoiding complex syntactic structures [17, 18].

The present study split Easy Finnish into two further variants, Basic Easy and Easiest Easy (see Table 2 for their detailed comparison). **Basic Easy Finnish** is the most common simplified language variant in Finland, defined as: “… a form of language in which vocabulary, language structures and contents are modified to be more readable and understandable than in standard language. It is intended for people who have difficulties reading or understanding standard language” [26]. As **Easiest Easy Finnish** is in its initial stages, no specific official guidelines have yet been provided for producing it. Yet some earlier studies suggest that even less than 100 simple words could be used for explaining complex words and concepts [20, 27].

**Table 2:** The upper part of the table shows the PRIME-MD PHQ two-question depression screen in English in three levels of linguistic complexity. The lower part shows the approximate difficulty levels of the Finnish language [27].

Standard Language (A)	Moderately simplified (B)	Most simplified (C)	
1. During the past month, have you often been bothered by feeling down, depressed, or hopeless? 2. During the past month, have you often been bothered by little interest or pleasure in doing things?	1. Think about your feelings in the last month. Have you often been worried about feeling down or depressed or hopeless? 2. Think about your feelings in the last month. Have you often been worried about having no interest in anything or not wanting to do anything?	Do you often think like this: 1. Nothing good ever happens to me. 2. I don’t want to do anything. 3. I feel bad.	
**Standard language**	**Advanced Easy Language** (or Plain language). For people with minor reading challenges. Approx. levels B1–B2 of the Common European Framework of Reference for Languages (CEFR).	**Basic Easy Language.** For people who are mainly able to read by themselves but have considerable problems with standard Finnish. Approx. levels A2–B1 of the CEFR. The most common variant of simplified Finnish.	**Easiest Easy Language.** For people with the most severe language and reading difficulties, who need maximally simplified language, and probably do not read by themselves. Approx. levels A1–A2 of the CEFR. Not used very much.

### Intralingual and interlingual translation

The moderately simplified Finnish version (B in Table 2) was produced and approved by professional Easy Language experts at the Finnish Center for Easy Language, Selkokeskus. The same experts also approved the most simplified version (C in Table 2), which was drafted by the authors of this paper. These simplified versions were trusted and taken into the study setting as they were produced by Selkokeskus. After producing the different Finnish versions (intralingual translation), we then ordered a professional translation of all three versions into five different languages (interlingual translation): Arabic, English, Farsi, Russian, and Swedish (the English versions in Table 2, the others in S1 Table). This gave us altogether 18 versions of the original screen: three complexity levels in six languages (S1 Table). Notably, for the standard English language version, we used the previously published standard language screen directly [23].

We explained the motivation for this translation task to all the translators, and briefly described the concept of Easy Language. They were also allowed to search for PRIME-MD PHQ two-question depression screens in their target language if such was available. For both Arabic and Russian, two independent translations were ordered, from which one was chosen for the next step of the study on the basis of backtranslation.

### Interpreters’ survey

A survey was conducted among professional interpreters (hereafter called participants) to characterize their views on the different language versions, in particular with respect to their usefulness in different health care contexts. The survey was conducted using a Google Form platform, which included six sections with multiple subsections, altogether 15 questions. The different language versions were randomly named A, B and C so that the survey participants remained blind to the original version categories. All the instructions and survey questions were given in Finnish, but the screen versions were delivered in all five target languages. An English translation of the entire questionnaire is available in S1 Questionnaire.

All the participating interpreters were recruited via a well-known and trusted Finnish interpreting company in April 2021 and were paid their usual hourly rate for answering the questionnaire. We emailed the interpreting company, informing them of our research on Easy Language at the University of Helsinki, and requesting “a total of ten public service interpreters specialized in health care for a pilot study. We need two interpreters for all the following language pairs: Finnish–Swedish, Finnish–Russian, Finnish–English, Finnish–Arabic and Finnish–Persian”. We also gave the company a list of names of interpreters who were familiar with our project and could thus not participate in the study. All the ten professional interpreters suggested by the interpreting company participated in our pilot study. Two interpreters were recruited per each language (total N = 10). In the background questions, the interpreters described their experience as a great deal (N = 7) or some (N = 3) experience in interpreting in health care. These were the only demographic questions the interpreters had to answer (see S1 Questionnaire). The participants in the survey setting had not been involved in the earlier steps of the study. There were no time limits for filling in the form. No ethical issues needed consideration, nor any ethical approvals were needed for this pilot study, as no clients or patients were involved. The authors did not have access to information that could identify individual participants during or after data collection.

## Results

The most consistent finding of the questionnaire was that the professional interpreters found the moderately simplified and the most simplified language versions beneficial for successful interpretation between languages. They also considered the most simplified versions especially useful for clients with more than one linguistic challenge.

At the beginning of the survey, nine out of ten interpreters perceived the interpretation task as easy or somewhat easy, even when they were shown the Standard Finnish version. Despite this perceived ease, however, eight (80%) of them still indicated that they would prefer to use either of the simplified versions when translating for their clients.

Next, we wanted to assess whether the language competence of the target person (client of the interpreter) would affect their choice. The participants were given all three Finnish versions, and were asked to choose their favorite for clients with different profile descriptions (see Table 3). Most of the clients presumably had other linguistic challenges in addition to their need for cross-language interpretation.

**Table 3:** Answers to question “Now you as an interpreter can choose form A, B or C. Which option would you choose if your client was…”.

Other characteristic than the need for interpreting between languages	Standard Finnish (A)	Moderately Simplified (B)	Most Simplified (C)
An elderly person with dementia	1	1	8
A seven-year-old child	1	1	8
An illiterate adult	1	2	7
A person for whom the language of interpretation is not their mother tongue	2	1	7
An adult with a cognitive disability	1	3	6
A working adult who has been involved in a serious accident	2	6	2
A young computer science student	5	5	0
Total votes for each simplicity version	13	19	38

The results show that the moderately simplified and the most simplified versions were generally favored, but that they did not suit everybody. For instance, an elderly person with dementia was considered to benefit from the most simplified version, whereas a young student was believed to gain more help from the standard version. Hence, the moderately simplified and the most simplified were more useful if the client and doctor spoke different languages and if the client’s language was compromised in any way.

In order to compare all three simplified versions of both the source and target languages, the participants were asked to rate all three translations in both Finnish and their target language (i.e., each participant was shown six versions). We asked for their assessment of both their interpretation task and of their clients’ understanding. In the analysis of the results, we transferred the answers into points: very poor > minus 2, fairly poor > minus 1, fairly good > plus 1, very good > plus 2. Each language option thus received points from the interpreters. These results are presented in Table 4.

**Table 4:** Results of assessment of usefulness of various language and complexity versions for interpreting and clients’ understanding. The question was “How useful are the options in terms of understanding?”.

	very good / fairly good	very bad / fairly bad	total score
Moderately simplified Finnish	13	0	13
Moderately simplified target language	13	0	13
Most simplified target language	14	1	13
Most simpified Finnish	15	2	13
Standard Finnish	12	2	10
Standard target language	9	3	6

In concordance with the results above, the moderately simplified versions and the most simplified versions were rated more highly than the standard language versions. Notably, the standard language versions were not considered very useful from the interpreting viewpoint. Likewise, most (7 out of 10) of the participants claimed they would choose the moderately simplified or the most simplified versions for their client situations. In the free-form feedback, the participants reported that they chose the simpler version due to its clarity and simplicity. The somewhat counterintuitive finding that standard Finnish was rated higher than standard target language would require further studies.

Finally, the participants were asked to justify nine statements about the different aspects of interpretation work. As Table 5 shows, there was a considerable consensus on certain arguments. For instance, nine out of ten participants felt that *the interpreter can effectively interpret the standard language Finnish screen at the doctor’s office*––which likely reflects their professional self-confidence. Conversely, nine out of ten participants felt that *the Easy Finnish version helps in interpreting the screen*––which corroborates the earlier results. Moreover, the majority (8 out of 10) of the participants said *there should be an Easy Language version of the screen in the client’s own mother tongue*. As the interpreters were confident about their own skills, this response likely implies that they feel their clients would benefit from simpler language versions. In accordance with this interpretation, most of the participants (7 out of 10) also felt that *the Easy Language versions are suitable for all clients*.

**Table 5:** Answers from statement justification task. Considerable consensus among participants (N = 10) on certain statements, but some contradiction on others.

	Yes	No	I don’t know
The Easy Finnish version helps in interpreting the screen.	9	0	1
The interpreter can effectively interpret the standard language Finnish screen during a doctor’s appointment.	9	0	1
There should be an Easy Language version of the screen in the client’s own mother tongue.	8	1	1
The Easy Language versions are suitable for all clients.	7	1	2
The screen should be a standard language version in the client’s own mother tongue.	6	0	4
The screen should be in standard Finnish.	6	1	3
It is easy for the interpreter to know whether or not the client has understood the content of the screen.	5	3	2
The interpreter should have all the different versions of the screen available.	4	3	3
The Easy Language version can be a disadvantage when interpreting.	0	6	4

## Discussion

Our results provide proof of concept that medical screening surveys can be successfully translated into versions with different linguistic complexity, and that these versions can be translated across a wide range of languages. Most importantly, the findings show that the professional interpreters consistently preferred simplified language versions when they had to choose the optimal source and/or target language versions for their clients (patients) with varying levels of linguistic compromise. Our findings are fully in line with those of many previous studies showing the importance of language for successfully conducting questionnaires (e.g., [5]), as well as the benefits of simplifying language in psychometric settings in particular (e.g., [28]). In contrast to the simplification level seen in earlier studies (e.g., [14–16]), our results suggest even greater level of language simplification for better comprehensibility. Here we expand on these prior findings by showing that these intuitively conceivable ideas can be generalized to apply to cross-language contexts, and the benefits are even seen by people such as professional interpreters, who themselves are highly competent in the studied languages.

Previous studies of Easy German have shown how most simplified language can be beneficial for people whose serious linguistic challenges are known [17]. For people with less serious challenges, the less simplified or even the standard language variants were preferred [17, 29]. The language incompetence of a tested population is often unpredictable and calls for flexible and rapidly adapting solutions. This need can be met by producing simplified language versions, which can be offered to the tested individuals to ensure maximal understanding. Questionnaire-based psychometric assessments rely on language, and it is often implicitly assumed that the target individuals show sufficient linguistic competence for an unbiased assessment. This assumption is, however, in conflict with the wider literature and the everyday experience of how linguistic compromise is common among people who need the support of health or social care (e.g., [17, 30]). This conflict has led to a situation in which language *per se* has become a stumbling block to reliable assessment, and it calls for creative solutions in everyday practice [31].

The language of psychometric assessments should be at least simple and clear enough to be understood by all people with any level of language competence in the target population. Here, we showed that it is possible to simplify the language in psychometric assessments by using a structured and linguistically sound approach. We also showed that these simplified language versions are favored by the majority of professional interpreters.

Our study further demonstrated that the simplified versions of a psychometric assessment can be successfully translated into all the tested target languages, and again, the professional interpreters favored the simplified versions over the standard version, which has conventionally been used in medical contexts. The grammatical constructions and information structure were adopted for both the moderately and most simplified versions according to Easy Language guidelines. For the most simplified version, the simplification process was based on the universal vocabulary of Minimal Language [32], using semantic primes as the building blocks for the linguistically easiest psychometric screen possible.

Why is simplification needed in psychometric assessments? The phenomena to be assessed are typically complex and abstract, and conversely, they often need description with complex, abstract, and culturally varying vocabulary. This has created a culture in which professionals, who themselves are well versed in the given phenomenology, may favor complexity as a means of producing “succinct expressions”. However, the understanding of these concepts and terms may significantly vary among those outside the inner circle of experts, including other health care workers and those to be assessed by the given psychometric tests, and this may go unnoticed. The often elusive nature of the tested psychological or social phenomenology *per se* is likely to introduce significant ambiguity to the success of testing.

For people who only speak one language, it might be difficult to understand how problematic directly translating mental health screening instruments can be. As well as disorders, depression and anxiety are also complex feelings, and many studies of both lexical semantics and psycholinguistics have shown that feelings may vary from one language to another. For example, it has been argued that people who do not know Japanese may not recognize the emotion *amae* [33], or that people who do not speak German may not feel the same *Angst* as German-speakers do [34]. Direct translation issues have also caused problems in emergency communication, and detecting translation problems and tailoring communication systems to the needs of the entire community at risk has been deemed essential [35]. Similar direct translations complications may be expected in mental health screening.

The comprehensibility of language could and should be routinely assessed when producing material for assessing mental health. Our study shows how interpreters’ implicit perceptions, a rarely considered resource, can be exploited to assess the suitability of different language versions for target populations. Professional health care interpreters are a potentially valuable informant group because they have practical experience in performing *ad hoc* translations of psychometric screens, and they have unique insights into the grass-root levels of using psychometric instruments. Unlike most doctors or patients, interpreters, as language professionals, are “linguistically tuned”, making them more competent to analyze situations in which the language used may lead to misunderstandings.

Our work raises an interesting alternative to or complementary component of questionnaire production and the translation process: It has been customary to carry out resource-intensive, formal validation studies of questionnaire translations that are direct translations from the standard language versions. Our work suggests, however, that intra-language translations, i.e., simplification of the questionnaires in the source language itself could also offer significant added value. Conversely, inter-language translations should perhaps be considered for different levels of simplicity, unless a specific version is perceived as the most useful by a reference group. In other words, the acceptability and comprehensibility of the language should become an integral part of the development process. The lack of such “user experience” aspects in current questionnaire development practices does, indeed, challenge the ecological validity of the process, despite perhaps satisfying formal testing requirements. Further study settings could also include migrant patients explaining how they understand the questions in their own words and health professionals to assessing the usefulness of the simplified versions. Our results suggest that simplified versions of the depression screen could be used as alternative variants as needed, case by case, even without formal validation processes, like it is already actually happening in oral communication in practice. Of course, we as linguists are eagerly looking forward seeing formal validation processes carried out by e.g., psychometric researchers.

Our present results also support the generation of simplified language versions of other types of psychometric tests, such as those for anxiety and stress. A multilingual open database could help construct simplified versions of psychometric instruments, as much of the grammatical and informative content in the psychometric instruments may be shared across different kinds of tests. Such a database could provide direct solutions for situations in which official standardized screens fail to work [31], and importantly, it would enhance interdisciplinary collaboration between linguistics and the many disciplines that rely on psychometric assessments. Such efforts could be particularly useful for supporting large international programs, such as PROMIS, a multinational initiative developing new ways to measure patient-reported outcomes [36]. Interdisciplinary collaboration would also help develop questionnaires drawing on a theoretical understanding of the components of the condition.

## Limitations

The current study had a few potential limitations. First, it was conducted in the Finnish language, and the linguistic simplification was first made in Finnish. Only the Swedish version was confirmed as following the Easy Swedish guidelines: The simplified versions of the other languages (Arabic, English, Farsi, Russian) were produced by professional translators from the source language. Second, we supposed, but did not formally assess, the formal Easy Language expertise of these translators. Third, we are aware that languages such as Arabic have many dialogs even within the same country, and it may be that no single language variant is commonly understandable to all native speakers. Fourth, the sample size of ten interpreters (two for each language) was small and as we did not elicit their detailed demographics, we cannot report them in this paper. As regards the participating interpreters’ language skills, qualifications, and expertise, we trusted the interpreting company who supplied them in this issue. Fifth, the study setting could have minimized the risk stereotyping of the customers/patients. Some cognitive interviewing would have been beneficial to explore participants’ reasoning in choosing e.g., which versions would work best with which service users. The results of our pilot study should thus be seen as proof of concept only, and further studies are definitely needed.

## Conclusion

In conclusion, assessment of mental health relies heavily on language, which can be significantly simplified for better comprehensibility. Here, we provide proof of concept for the idea that professional interpreters can be used to assess the utility of such intra-language and inter-language translations. The ultimate proof needed for wider clinical uptake of such versions will come from the extent to which hands-on workers consider language simplifications can bring “perceived added value” to everyday health care situations.

## Supporting information

S1 Table PRIME-MD PHQ two-question depression screen in three levels of complexity. The moderately simplified and most simplified versions were first made for Finnish, and then translated to other languages in the study.
(DOCX)

S1 Questionnaire An approximate English translation of the questionnaire used in the study setting. The original questionnaire was in Finnish.
(DOCX)

S1 Appendix Original questionnaire data in Finnish. (XLSX)
