# Supplementary material for: Simplifying phrases in depression screens: Interpreters’ views on usefulness in six languages
Source: PLoS One. 2023 Dec 8;18(12):e0292365. doi: 10.1371/journal.pone.0292365 (PMC10707561; doi:10.1371/journal.pone.0292365)
Supplement: S1 Table — The moderately simplified and most simplified versions were first made for Finnish, and then translated to other languages in the study. (DOCX) [file pone.0292365.s001.docx]

**S1 Table. PRIME-MD PHQ two-question depression screen in three levels of complexity.** The moderately simplified and most simplified versions were first made for Finnish, and then translated to other languages in the study.

| Language | Standard Language (A) | Moderately simplified (B) | Most simplified (C) |
| --- | --- | --- | --- |
| Finnish | 1. Oletko viimeisen kuukauden aikana usein ollut huolissasi tuntemastasi alakulosta, masentuneisuudesta tai toivottomuudesta?    2. Oletko viimeisen kuukauden aikana usein ollut huolissasi kokemastasi mielenkiinnon puutteesta tai haluttomuudesta? | 1. Ajattele tunteitasi viimeisen kuukauden aikana.  Oletko ollut usein huolestunut siitä, että olosi on   - alakuloinen - tai masentunut - tai toivoton?     2. Ajattele tunteitasi viimeisen kuukauden aikana.  Oletko ollut usein huolestunut siitä, että   - sinua ei kiinnosta mikään - tai et halua tehdä mitään? | Ajatteletko sinä usein näin:  1. Minulle ei tapahdu mitään hyvää.  2. Minä en halua tehdä mitään.  3. Minusta tuntuu pahalta. |
| English | 1.During the past month, have you often been bothered by feeling down, depressed, or hopeless? YES/NO    2.During the past month, have you often been bothered by little interest or pleasure in doing things? YES/NO | 1. Think about your feelings in the last month.  Have you often been worried about feeling   - down - or depressed - or hopeless?     2. Think about your feelings in the last month.  Have you often been worried about   - having no interest in anything - or not wanting to do anything? | Do you often think like this:  1. Nothing good ever happens to me.  2. I don't want to do anything.  3. I feel bad. |
| Swedish | 1.Har du under den senaste månaden ofta känt dig nere, känt dig deprimerad eller känt att framtiden ser hopplös ut?    2. Har du under den senaste månaden ofta haft minskat intresse eller minskad lust att göra saker som du vanligen tycker om? | 1. Tänk på hur du har känt dig den senaste månaden.  Har du ofta känt dig   - ledsen - eller deprimerad - eller förtvivlad?   2. Tänk på hur du har känt dig den senaste månaden.  Har du ofta känt att   - ingenting intresserar dig - det inte finns något du vill göra? | Tänker du ofta så här?  1.Det händer inget bra i mitt liv.  2. Det finns ingenting som jag vill göra.  3.Jag känner någonting  dåligt. |
| Russian | 1. В течение последнего месяца часто ли вас беспокоило чувство подавленности, депрессии или безнадежности?  2. В течение последнего месяца часто ли вас беспокоило отсутствие интереса или желания что-либо делать? | 1. Подумайте о своих чувствах за последний месяц. Часто ли вас беспокоило то, что вы чувствуете себя  • подавленно  • или депрессивно  • или безнадежно?    2. Подумайте о своих чувствах за последний месяц. Часто ли вас беспокоило то, что  • вам ничего не интересно  • или вы не хотите ничего делать? | Часто ли вы думаете так:  1. Со мной не случается ничего хорошего.  2.Я не хочу ничего  делать.  3.Я чувствую себя плохо. |
| Arabic | 1. خلال الشهر الماضي، هل كنت قلقًا بشأن الشعور بالحزن أو الاكتئاب أو اليأس؟  2. في الشهر الماضي، هل كنت في الغالب قلقًا من تجربة الأشياء الممتعة أو عدم الرغبة بذلك؟ | 1. فكّر في مشاعرك خلال الشهر الماضي.  هل كنت قلقًا بشأن شعورك بـ   - الحزن - أو الاكتئاب - أو اليأس؟   2. فكّر في مشاعرك خلال الشهر الماضي.  هل كنت في كثير من الأحيان قلقًا بشأن   - عدم اهتمامك بأي شيء - عدم رغبتك بفعل أي شيء؟ | هل تفكر هكذا كثيرًا:  1. لن يحدث شيء جيد بالنسبة لي.  2. لا أريد أن أفعل أي شيء.  3. أشعر بشعور سيء. |
| Farsi | 1. آیا در یک ماه گذشته اغلب نگران اندوه، افسردگی یا نا‌امیدی بوده‌اید که احساس کرده‌اید؟    2. آیا در یک ماه گذشته اغلب نگران کمبود علاقه و دلبستگی یا بی‌میلی بوده‌اید که تجربه کرده‌اید؟ | 1. به احساسات خود در یک ماه گذشته فکر کنید. آیا اغلب نگران این بوده‌اید که وضع و حال شما  ·   اندوهگین است  ·   یا افسرده است  ·   یا ناامید است؟    2. به احساسات خود در یک ماه  گذشته فکر کنید. آیا اغلب نگران این  بوده‌اید که  · هیچ چیزی برایتان جالب نیست  · یا میل ندارید هیچ کاری انجام  دهید؟ | آیا شما اغلب چنین فکر می‌کنید؟  1. هیچ چیزی خوبی برای من اتفاق نمی‌افتد.  2. من میل ندارم هیچ کاری انجام دهم.  .3 من احساس بدی دارم. |
