# Supplementary material for: Simplifying phrases in depression screens: Interpreters’ views on usefulness in six languages
Source: PLoS One. 2023 Dec 8;18(12):e0292365. doi: 10.1371/journal.pone.0292365 (PMC10707561; doi:10.1371/journal.pone.0292365)
Supplement: S1 Questionnaire — The original questionnaire was in Finnish. (DOCX) [file pone.0292365.s002.docx]

**S2 Questionnaire. An approximate English translation of the questionnaire used in the study setting.** The original questionnaire was in Finnish.

**Part 1**

The purpose of this survey is to study a screen for diagnosing depression, interpreting, and easy language. There are no right or wrong answers to the questions. Answer as you see fit. For more information on the study, contact NN. By completing this survey, you agree to the answers being used in the study. Thank you for participating!

**Part 2**

Imagine you are interpreting at a doctor’s office. The doctor uses a form to determine whether the patient has depression. The form has two questions:

1.During the past month, have you often been bothered by feeling down, depressed, or hopeless?

2.During the past month, have you often been bothered by little interest or pleasure in doing things?

What do you think of this interpreting task?

- The form is easy to interpret so that the client understands what it is about
- The form is reasonably easy to interpret so that the client understands what it is about
- The form is quite difficult to interpret so that the client understands what it is about
- The form is difficult to interpret so that the client understands what it is about

What questions in the form do you find difficult to interpret?

- Some words are difficult to interpret
- Some structures are difficult to interpret
- The whole thing is difficult to interpret
- Nothing is difficult to interpret

If any point is difficult to interpret, please tell us more about it here.

**Part 3**

In the following, you will see three different versions of the same form. The doctor wants to know if the client is depressed. In addition to the original form (A), there are two new versions (B and C).

What do you think would be the best version for interpreting?

Option A

Option B

Option C

Please justify your choice. Why do you think the option you have chosen is the best?

Now you as an interpreter can choose form A, B or C. Which option would you choose if your client was:

An elderly person with dementia

An adult with a cognitive disability

A young computer science student

An illiterate adult

A working adult who has been involved in a serious accident

A person for whom the language of interpretation is not their mother tongue

A seven-year-old child

**Part 4**

Next you will see three different versions (A, B, C) of the same form in both Finnish and the target language.

Evaluate how good the versions are in terms of interpreting and client understanding (very good, fairly good, fairly poor, very poor).

Finnish option A

Finnish option B

Finnish option C

Target language option A

Target language option B

Target language option C

If you could only choose one version to help with interpreting, what would it be?

Finnish option A

Finnish option B

Finnish option C

Target language option A

Target language option B

Target language option C

Please briefly justify your choice. Why did you choose this particular version?

Choose what you consider the best option for the following statements (Yes, No, I don’t know). All the statements apply to the use of the depression screen during a doctor’s appointment.

The screen should be a standard language version in the client’s own mother tongue.

The screen should be in standard Finnish.

The interpreter can effectively interpret the standard language Finnish screen during a doctor’s appointment .

There should be an Easy Language version of the screen in the client’s own mother tongue.

The interpreter should have all the different available versions of the screen.

The Easy Finnish version helps in interpreting the screen.

The Easy Language versions are suitable for all clients.

The Easy Language version can be a disadvantage when interpreting.

It is easy for the interpreter to know whether or not the client has understood the content of the screen.

**Part 5**

Finally, we ask a few more general questions about interpreting related to forms.

Evaluate how often you need to interpret some kind of form in a health care setting.

Every time

About every other time

Less often than every other time

Almost never

What do you think about interpreting forms from Finnish to the target language?

The forms are usually easy to interpret

The forms are usually quite easy to interpret

The forms are usually quite difficult to interpret

The forms are usually difficult to interpret

What do you think makes interpreting these forms difficult?

**Part 6**

The answers will be processed anonymously. In order to analyze the answers, we need information about your interpreting experience.

How much experience do you have in interpreting in health care settings?

I have no experience interpreting in health care.

I have little experience in interpreting in health care.

I have some experience interpreting in health care.

I have a lot of experience interpreting in health care.

Would you like to say something more about the topic? If so, please do so here.
